# Synthesis, Mesomorphic and Computational Characterizations of Nematogenic Schiff Base Derivatives in Pure and Mixed State

**DOI:** 10.3390/molecules26072038

**Published:** 2021-04-02

**Authors:** Laila A. Al-Mutabagani, Latifah A. Alshabanah, Hoda A. Ahmed, Hafsa H. Alalawy, Mayada H. Al alwani

**Affiliations:** 1 Chemistry Department, College of Science, Princess Nourah bint Abdulrahman University, Riyadh 11671, Saudi Arabia; Laalmutbagani@pnu.edu.sa (L.A.A.-M.); laalsabanah@pnu.edu.sa (L.A.A.); 2 Department of Chemistry, Faculty of Science, Cairo University, Cairo 12613, Egypt; hafsahamdy@gmail.com; 3 Chemistry Department, College of Sciences, Yanbu, Taibah University, Yanbu 30799, Saudi Arabia; tu4053550@taibahu.edu.sa

**Keywords:** azomethine liquid crystals, nicotinate derivative, nematic phase, geometrical structure, DFT, optimized structure, binary phase diagram

## Abstract

Homolog series based on three aromatic rings bearing terminal alkoxy chain of various lengths named 4-(4-(alkoxy)phenylimino)methyl)phenyl nicotinate (**An**) were synthesized. The alkoxy-chain length changed between 6, 8 and 16 carbons. Mesomorphic and optical properties were carried out via differential scanning calorimetry (DSC) and polarized optical microscopy (POM). Elemental analyses, FT-IR and NMR spectroscopy were carried out to elucidate the molecular structures of the prepared derivatives. Mesomorphic results indicated that all the synthesized homologs (**An**) are monomorphic possessing the nematic (N) phase enantiotropically with wide thermal stability. Computational simulations were measured via density functional theory (DFT) theoretical calculation tool. The estimated thermal and geometrical parameters are in agreement with the experimental data. By discussing the estimated parameters, it was found that the molecular architecture, dipole moment and the polarizability of the investigated compounds are highly affected by the length of the attached terminal flexible chain and the location of the nitrogen atom in the other terminal aromatic ring. Binary phase diagrams of two corresponding homologs with different proportionating terminals were constructed, and their binary phase physical properties were discussed in terms of the temperature range and stability of the N phase.

## 1. Introduction

Nowadays, liquid crystals (LCs) based on organic derivatives prove to have broad areas of industrial applications as optical technology and photoconductors [1,2,3]. Structural–characteristic relationships are important approaches to prepare compatible geometrical shapes to achieve the essential properties for recent technological applications [4,5,6,7]. Selection of attached substituents, flexible chains and mesogenic spacers are important criteria in the designing of proper thermotropic LCs for applications. Modifications in the molecular architectures lead to change in the mesomorphic behavior and play important roles in the formation, kind and thermal stability of the produced mesophase [8,9,10].

Insertion of heterocyclic pyridine ring in the LCs’ skeletons strongly impacts their polarizability and/or polarity as well as their geometric structures. Consequently, it extensively affects the phase transition temperature, types of mesophase and other physical and geometrical parameters essential for better properties of the LC materials [11]. Incorporation of Schiff base and ester linking groups widely improves many properties of LCs. This could be attributed to their rigidity that enhances the stability of LC mesophases [12,13]. These modifications in the properties of the LCs may be beneficial for mesomorphism as well as the physical properties necessary for technical uses. Schiff base linkages have been broadly employed in the preparation of numerous LC derivatives [14,15,16,17]. Most reports are focused on Schiff bases since the discovery of nematogenic 4-methoxybenzylidene-4′-butylaniline at room temperature [18]. Recently, several thermotropic Schiff base/ester liquid crystals have been documented [19,20,21].

Today, the designing of LC compounds according to theoretical prediction has attracted high interest [22,23,24,25]. Understanding many optical parameters requires increased knowledge about the molecular geometries and energies of molecular orbitals of the mesomorphic compounds. Recently, density functional theory (DFT) has become an effective tool for its excellent performance and computational data being consistently correlated with the experimental findings [22,23,24,25,26].

Decrement of the melting temperatures of the LCs and broadening their mesophase range are important goals for many practical applications. One way to achieve a low melting point is the mixing of compounds possessing various molecular shapes and characteristics in binary systems [26,27,28,29,30,31,32,33]. The mesomorphic properties are greatly modified upon the mixing of individual components. These components may be symmetric, with identical mesogenic moieties; or non-symmetric, with different mesogenic linkers. In both cases, the specific interactions between the two mesogenic compounds do lead to a significant variation in the mesophase behavior of such materials [34,35,36,37,38,39].

Recently, our team has centered its interest on the computational simulations of new synthesized LC materials to experimentally illustrate their mesophase properties in terms of theoretical estimations [26]. These investigations were focused on the obtained mesomorphic transition results and the evaluated theoretical calculations correlations for investigated compounds. The geometrical conformations of LCs depend on their structural shapes that play an essential role in the stability and formation of their mesophases. Moreover, it has been found that the possible orientation of hetero-atoms in pyridines results in the modification of the existing functions, thus offering new geometrical properties to the organic compound [40]. Further, the length of flexible terminal chains plays important roles in the formation, stability, type and mesomorphic range of LC compounds. The mesomorphic molecules tend to be more oriented in a parallel arrangement as the length of the terminal moiety increases [41].

**Scheme 1:**
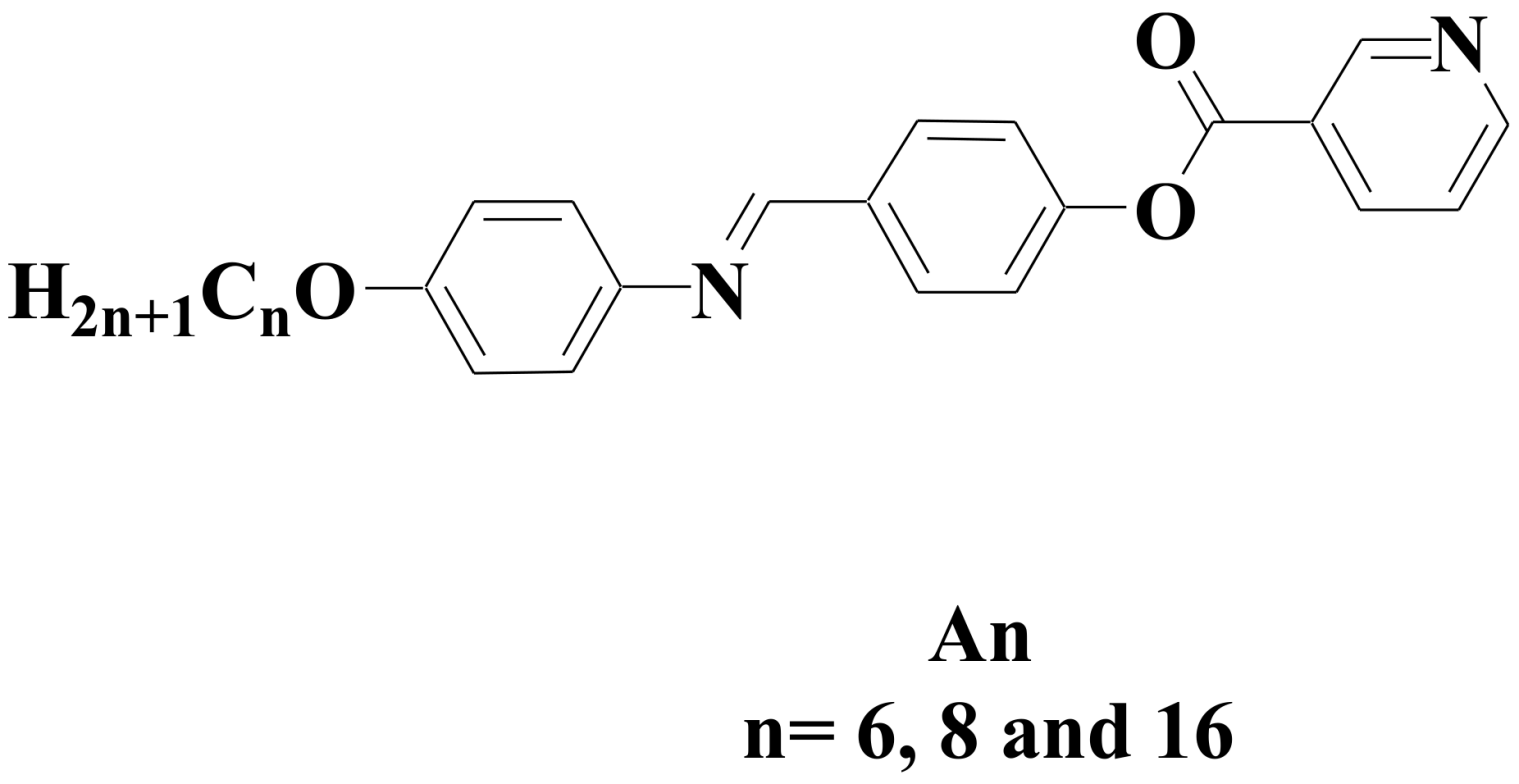
Investigated homologues series, **An**.

The goal of our investigation was to synthesize three-ring Schiff base/ester liquid crystals with a terminal heterocyclic ring (Scheme 1) and to investigate the effect of different proportionating of the terminal alkoxy chain length on the mesomorphic and optical behavior of synthesized derivatives. The evaluation was carried out by experimental and computational tools aiming to investigate their thermal and physical behaviors in pure and mixed states and to correlate the computational DFT simulations with the experimental variables data to explain the experimental results’ outcome.

## 2. Results and Discussion

### 2.1. Mesomorphic Characterizations

**Figure 1:**
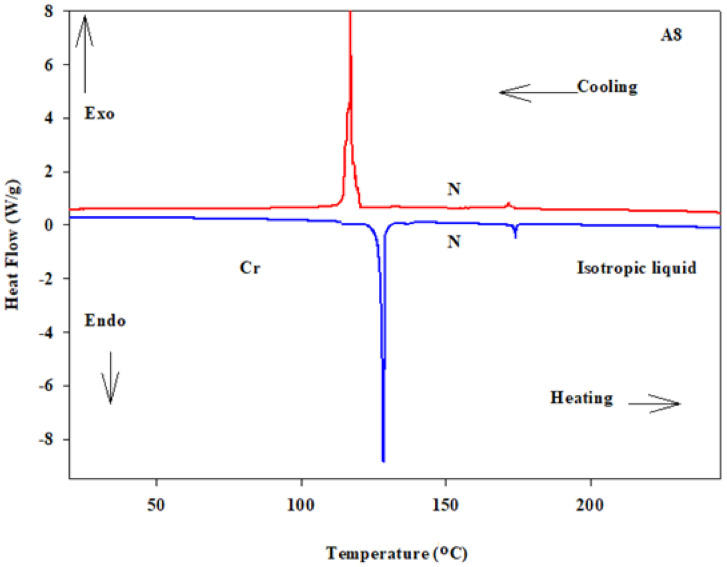
DSC thermograms of **A8** derivative upon second heating-cooling scan with a rate of 10 °C/min.

**Figure 2:**
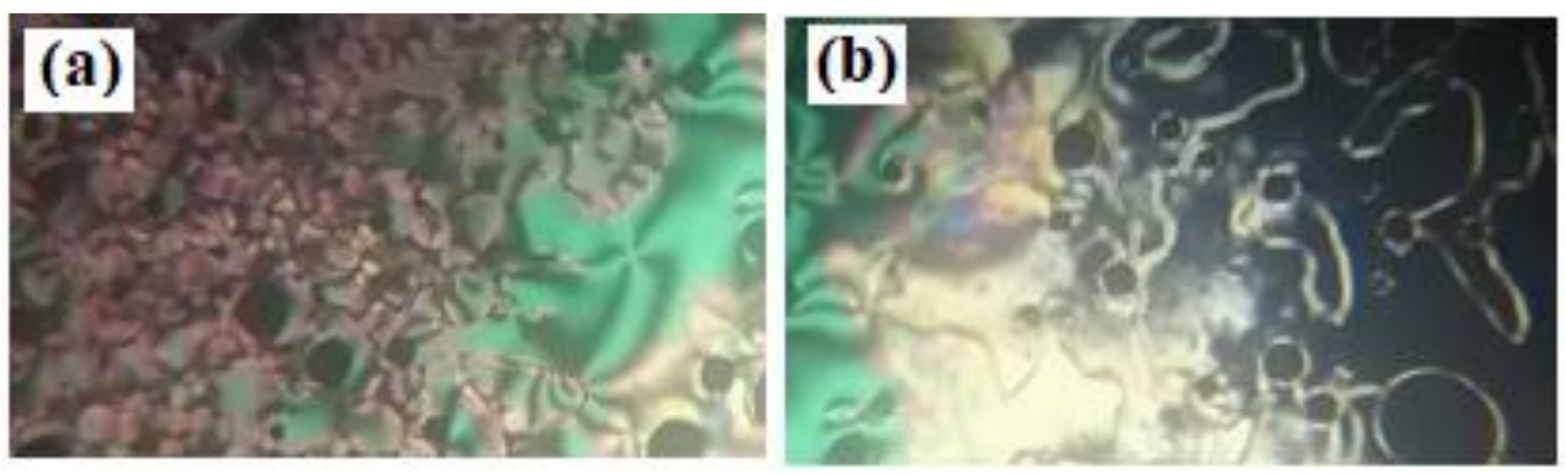
POM images of N mesophase upon heating of compound **A8** at (**a**) 158.0 °C and (**b**) 172.0 °C.

**Figure 3:**
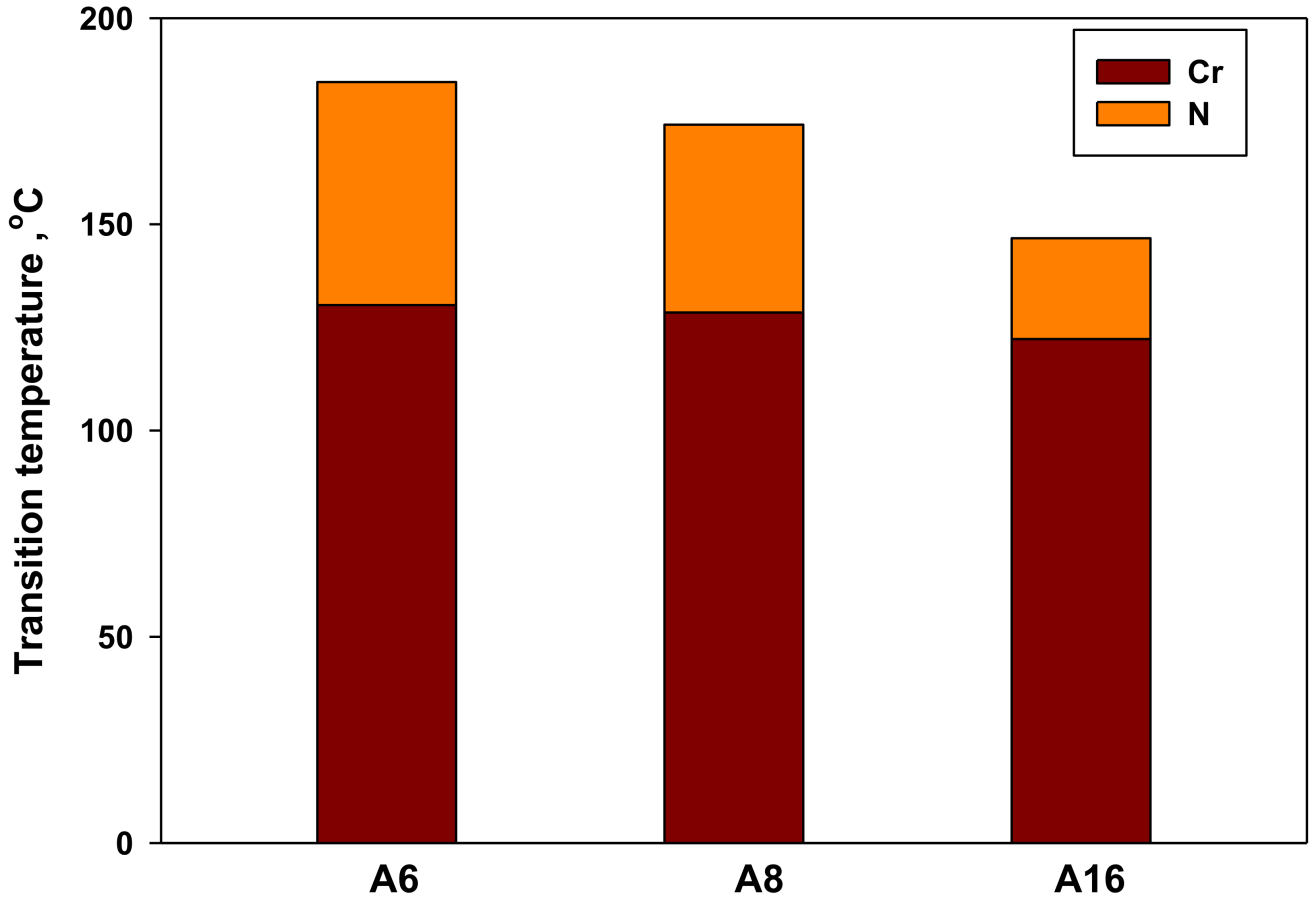
DSC transitions of synthesized groups **An**.

The phase transition temperatures, enthalpies and normalized entropies, as derived from DSC measurements, of synthesized homolog series **An** are collected in Table 1. DSC thermograms are represented in Figure 1 upon heating/cooling for **A8** derivative as an example and the others displayed in Figures S1 and S2 in supplementary data. Mesophase textures were identified by polarized optical microscopy (POM). POM images showed schlieren texture of nematic (N) phase covering all members of present group **An** upon heating and cooling (Figure 2). DSC measurements were performed for second heating scans to ensure the thermal stabilities of the investigated compounds. Moreover, DSC results were confirmed by the POM image observations. All members of the series showed to exhibit only two transition peaks upon heating and cooling cycles of DSC thermograms which are assigned to Cr-to-N (during heating) and N-to-Cr (upon cooling). In order to investigate the impact of terminal flexible alkoxy chain length on the mesomorphic properties of synthesized series, the graphical transition temperature relationship of DSC data is illustrated in Figure 3. Table 1 and Figure 3 reveal that all compounds of the homologs **An** are purely nematogenic possessing only the N phase enantiotropically. Moreover, there is, as usual, irregular change of their melting points with the increment of the terminal alkoxy chain length (n). It was previously documented that the N stability decreases gradually with n [36,37]. Thus, the homolog with the shortest alkoxy-chain (**A6**) exhibits the highest thermal nematic stability, 184.5 °C with a wide nematogenic temperature range of 54.1 °C. Meanwhile, the higher homolog **A16** possesses nematogenic stability and temperature range between 146.6 and 24.4 °C, respectively. The heterocyclic nicotinic moiety enables enhancement of the mesophase properties without any steric disruption, and consequently, N mesophases can still be observed covering all terminal lengths. Polarity of whole molecule also serves to enhance the optical transitions and other physical parameters. It was documented that [42] the type of mesophase and its stability are mainly dependent on the dipole moment of the mesogenic core of the molecule which varies according to the incorporated terminal groups. In general, the polarity and/or polarizability of the mesogenic moieties have a main role in improving the mesophase stability that will be proved in the theoretical calculation part. It could be seen also from Table 1 that all normalized entropies of transitions (**ΔS/R**) are of lower values, irrespective of the length of the alkoxy chain. This is in complete agreement with previous investigations [43].

**Scheme 2:**
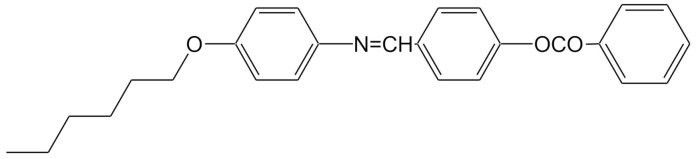
**B6** derivative.

**Scheme 3:**
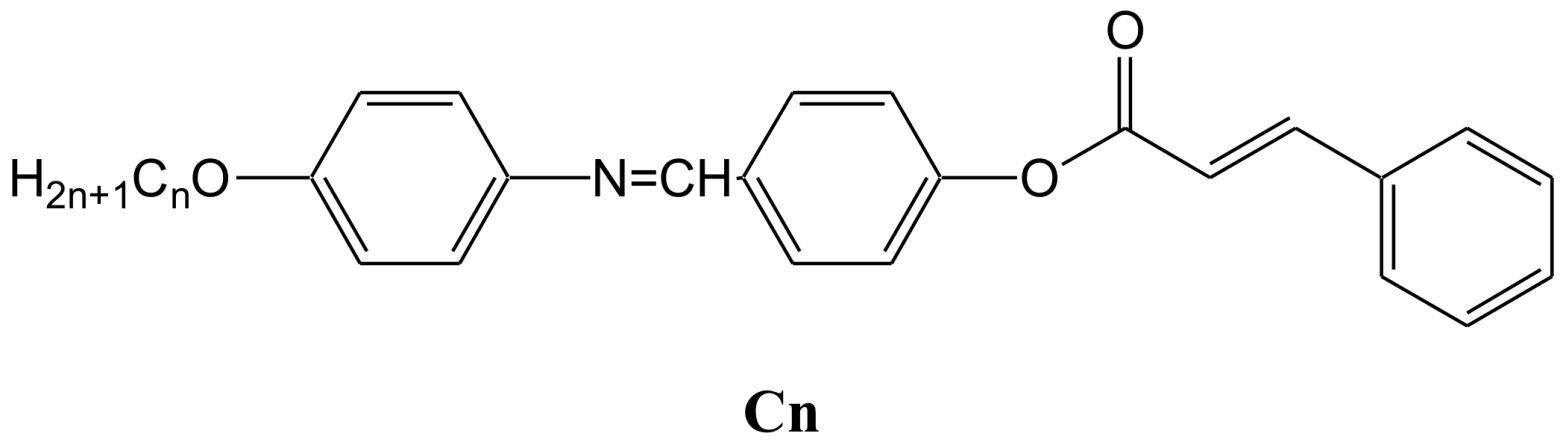
**Cn** homologues series.

A comparison is established between the present **A6** derivative and their corresponding isomer (**B6**, Scheme 2) bearing terminal benzene ring [16]. The results reveal that the type and stability of the observed mesophase are dependent on the enhanced dipole moment of the molecular mesogenic part which is mainly dependent on the hetero-ring moiety. In addition, the introduction of the pyridyl moiety rather than benzene ring disrupts the smectic A molecular packing and giving only the N phase. Another comparison is made between the present **An** series and their corresponding homologs **Cn** series (Scheme 3) [26] bearing terminal cinnamate moiety. The comparison revealed that the mesophase thermal stability of the formed N phase is dependent on the enhanced conjugation of C=C of the cinnamate group. Moreover, the insertion of the pyridyl group rather than the cinnamate moiety decrements by a small extent the nematic range and stability.

### 2.2. DFT Calculations

#### 2.2.1. Geometrical Simulations and Thermal Parameters

**Figure 4:**
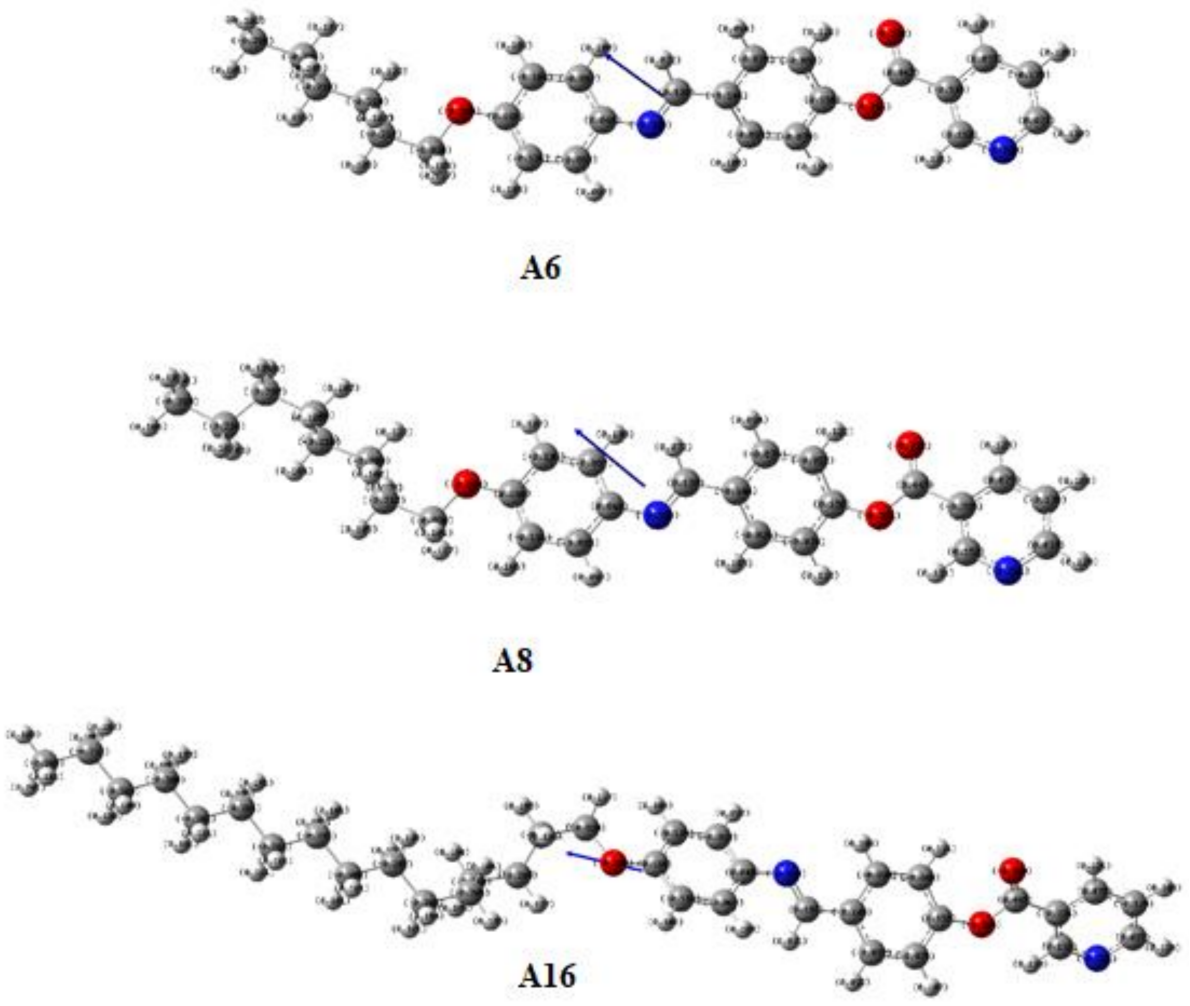
Atomic charges and dipole moment vector for the optimized structure of present series **An**, calculated at B3LYP/6-311g(d,p) level.

In order to study the effect of linking and terminal alkoxy chain groups on the mesomorphic properties of the present homologs **An**, computational calculations for each member of the investigated series were studied. The correlations were recognized between the quantum chemical parameters, derived by DFT theoretical calculations, and the experimental data variables for the synthesized series **An.** Calculations were estimated in the gas phase at B3LYP/6-311g(d,p) for predicting the optimum and most stable molecular geometries. Computational calculations were performed by Gaussian 09W package (Gaussian, Inc., Wallingford CT, USA) [44]. Data revealed that all compounds exhibit planar geometry with semi-linear molecular structure. Moreover, the length of terminal flexible chain highly impacts the structural geometry of the molecule (Figure 4).

The calculated total energy of the synthesized homologs series **An** and the other thermal parameters (thermal energy, enthalpy, Gibb’s free energy and entropy) at room temperature were estimated at the same level of theory and are summarized in Table 2. Moreover, other predicted parameters are collected in Table 3. Table 2 and Table 3 data show that the thermal and geometrical parameters predicted from DFT calculations are highly affected by the electronic conjugation of the linking groups. Moreover, the energy gap (∆E) between HOMO and LUMO levels is an indicator of the chemical reactivity of compounds. The lower its value, the more reactive the molecule would be. The predicted energy gap collected in Table 3 affirms the **A6** to be more reactive than others. It is also softer than others as the energy gap is inversely related to the softness. On the other hand, the lower ionization potential (I.E) calculated for the **A16** derivative indicates a more basic nature than others in the series.

Aspect ratio, polarity, polarizability, rigidity of the mesogenic cores and the molecular shape of the molecules, as well as the attached terminal chains, are essential parameters to enhance the type and thermal mesomorphic stability of the formed mesophase [45]. The competition between the intermolecular lateral and end-to-end interactions affects the mesomorphic properties of the resulted structure. The experimental and theoretical correlations revealed a pronounced increment of the mean polarizability with an increment of the length of terminal alkoxy chain (*n*) and a decrease of the nematic stability. As the chain length of the terminal group increases, the polarizability increases; this could be attributed to the increment in the aspect ratios. The space filling of the mesomorphic compounds increases as the aspect ratios of the molecular geometry increase, and this results in an enhancement of the polarizability. Moreover, the predicted polarizability shows an increment in its values as the nematic range (ΔT) decreases which is in agreement with our previous studies [26]. Furthermore, constant relation of the dipole moment and the N range and stability was observed. As the length of the alkoxy chain (*n*) elongates, the week van der Waals intermolecular attractions have an important role in destabilizing the observed N mesophase. The lower values of the estimated dipole moments allow the terminal aggregations to predominate more than the lateral one, which influences the N mesophase formation. In Table 3, the ionization energy and electron affinity, also calculated as I.E = −E_HOMO_ and E.A = −E_LUMO_, respectively, are included [46].

#### 2.2.2. Frontier Molecular Orbitals (FMOs)

**Figure 5:**
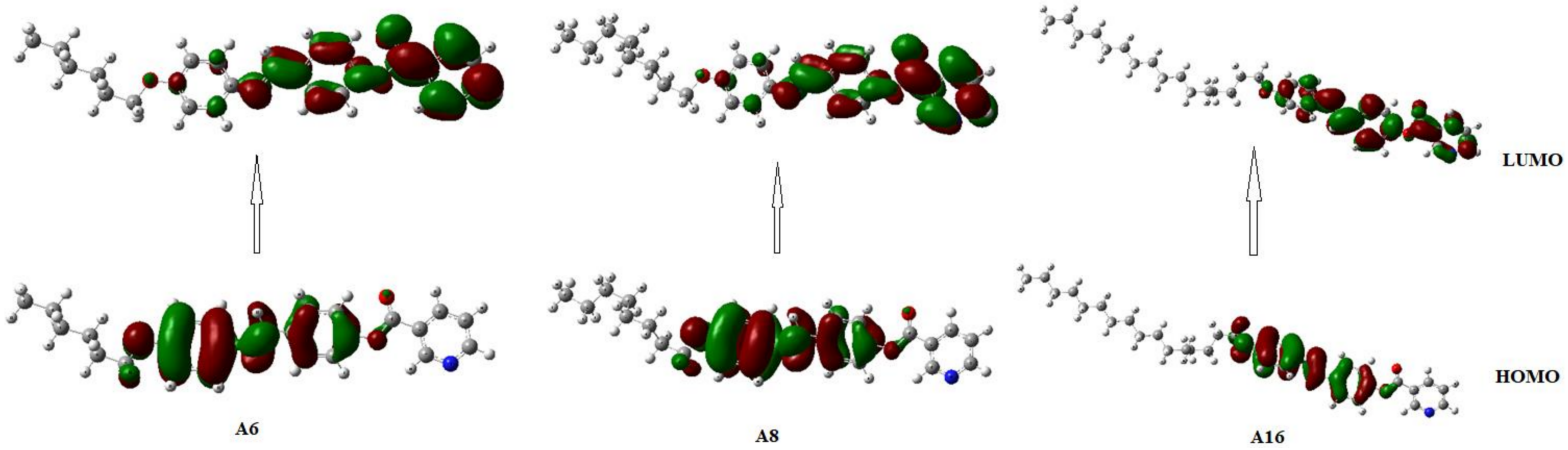
Estimated geometry for frontier molecular orbitals (FMOs) of the investigated homolog, **An**.

Optical investigations of the non-linear optical (NLO) materials are highly enhanced by the energy difference between the FMOs, HOMO (highest occupied molecular orbital) and LUMO (lowest unoccupied molecular orbital) [47]. The estimated ground state density surface plots for the FMOs of present groups **An** are simulated in Figure 5. The results of the FMO energy gaps are also collected in Table 3. As shown from Table 3 and Figure 5, the electron densities of the sites that contribute to the formation of the HOMOs and the LUMOs are localized on each of the azomethine linkage and the hetero-ring moiety. Moreover, the energy gap of FMOs is slightly dependent on the length of the alkoxy chain (*n*). It was reported that [26] the long terminal chain in addition to the conjugation within the mesogenic cores leads to a pronounced decrease in the FMO energy gap. Finally, the mesomorphic behavior of the rod-like mesogens develops the molecular–molecular interactions that essentially depend on the molecular geometry of prepared compounds, polarizability of the terminals and attached substituents, as well as the stereoelectronic characteristics of the whole molecule.

#### 2.2.3. Molecular Electrostatic Potential (MEP)

**Figure 6:**
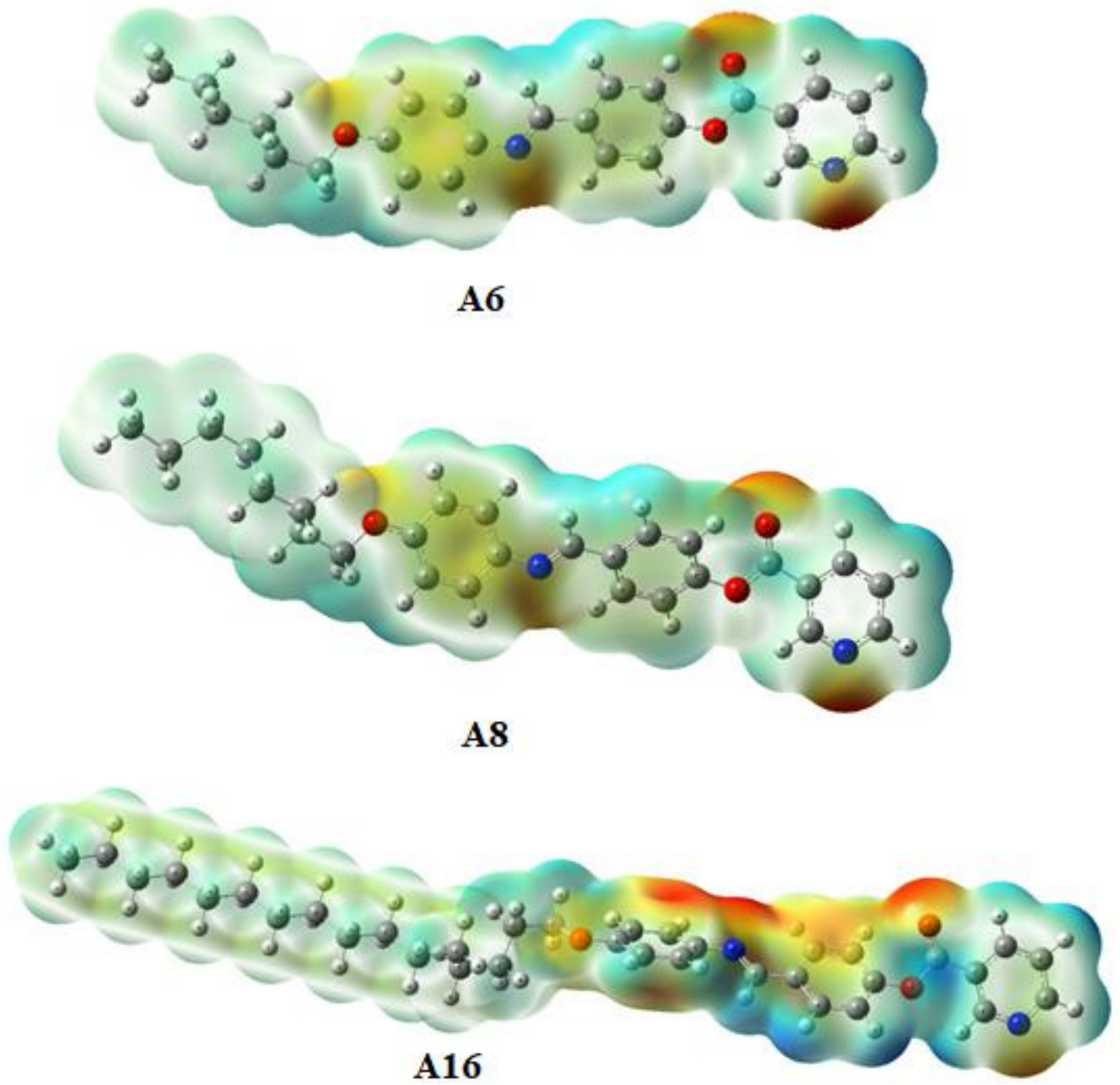
Molecular electrostatic potentials (MEPs) of the investigated group, **An**.

Charge distribution maps for the investigated group **An** were predicted under the same basis sets according to molecular electrostatic potential (MEP, Figure 6). The negatively charged atomic sites (red regions) were localized on the oxygen atoms and the nitrogen atom of the Schiff base linkage as well as the nitrogen of the hetero-terminal ring. While the alkyl chain showed the least negatively charged atomic sites (blue regions). As shown from Figure 6, the length of the terminal alkoxy group and the conformation of the attached aromatic ring are more effective on the orientation of the charge distribution map; this could affect the thermal nematic stability of the mesophase by alteration of the competitive interaction between end-to-end and side-side interaction. The measurements of theoretical charge distribution and the mesomorphic properties correlation were recently documented [26]. The alteration of the charge distribution on the whole molecules due to mesomeric interactions enhances the terminal aggregation to induce the N mesophase. Furthermore, the geometrical structure of the attached flexible chain affects the charge distribution map orientation. It was found that the longest terminal alkoxy group (**A16**) of the present homologs has the highest effect on the localization of the isoelectron density of the electron-rich and electron-deficient regions.

### 2.3. Binary Mixture

**Figure 7:**
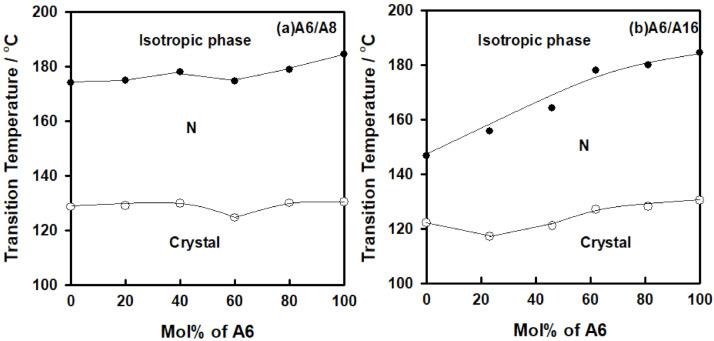
Binary phase diagram of (**a**) **A6**/**A8** and (**b**) **A6**/**A16**. “○” denotes crystal–nematic transition, and “●” denotes nematic–isotropic liquid transition.

From DSC measurements, binary phase diagram examples of terminally different lengthened alkoxy chains prepared from a combination of the homologs **A6** and **A8** as well as **A16** are presented in Figure 7a,b. As can be seen from these figures, the constructed binary mixtures from the shortest and longest terminals (**A6/A8** and **A6/A16**) showed to exhibit the N phase over the entire composition range of the binary mixtures. Figure 7a (**A6/A8** homolog) shows nearly ideal behavior as observed according to nematic stability for both derivatives. Meanwhile, a negative decrease from the ideality is observed for **A6/A16** homolog (Figure 7b). The nematic molecular association disruption in the longer chain length mixtures (**A6/A16**) is attributed to the non-similarity of the terminal flexible-chain length (*n* = 6 and 16), which is widely different between the two constructed derivatives. Figure 7a,b also show that the solid mixtures of **A6/A8** and **A6/A16** homologs possess eutectic melting points at 124.7 and 117.0 °C, respectively. Moreover, their eutectic compositions formed at 60 and 23 mol% of **A6** with N temperature ranges at the eutectic compositions ≈50.1 and 38.7 °C for **A6/A8** and **A6/A16** binary mixtures, respectively. Furthermore, the molecular geometry (which is affected by the stereo and/or mesomeric configurations) impacts the molecular-molecular interactions. It can be concluded that the terminal length of the flexible chain has a pronounced effect that controls both conformation and steric effect for pure and mixed states. This is consistent with our previous work [26,39].

## 3. Experiment

### Synthesis

**Scheme 4:**
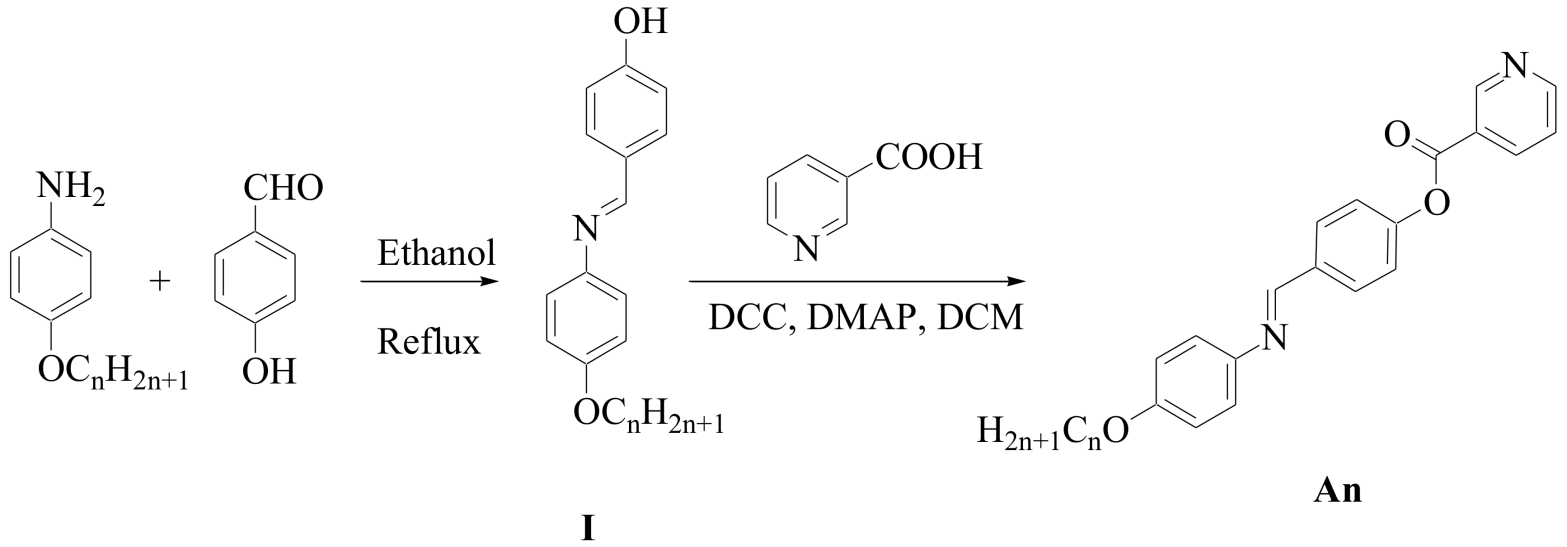
Preparation of *4-(4-(alkoxy)phenylimino)methyl)phenyl nicotinate* (**An**).

The investigated compounds **An** were synthesized according to the following Scheme 4.

*(Hexyloxy)phenylimino)methyl)phenyl nicotinate* (**A6**) [45]. Yield: 94.1%; mp. 131.0 °C, FTIR (ύ, cm^−1^): 2959–2833 (CH2 stretching), 1729 (C=O), 1591 (C=C), 1473 (C–OAsym), 1266 (C–OSym). ^1^H-NMR (400 MHz, CDCl3) δ (ppm) 10.08 (s, 1H, CH=N), 9.59 (s, 1H, pyr), 9.11–9.04 (m, 2H, pyr), 8.10–8.05 (m, 3H, pyr, ArH), 7.43 (d, *J* = 8.5 Hz, 2H, ArH), 7.01 (d, *J* = 8.1 Hz, 2H, ArH), 6.83 (d, *J* = 8.1 Hz, 2H, ArH), 9.93 (t, *J* = 6.2 Hz, 2H, CH_3_(CH_2_)_3_CH_2_CH_2_), 1.92–1.75 (m, 2H,CH_3_(CH_2_)_3_CH_2_CH_2_), 1.56–1.51 (m, 6H, CH_3_(CH_2_)_3_CH_2_CH_2_), 0.92 (t, *J* = 6.3 Hz, 3H, CH_3_(CH_2_)_3_CH_2_CH_2_). Elemental analyses: Found (Calc.): C, 74.58 (74.60); H, 6.50 (6.51); N, 6.94 (6.96).

*(Decyloxy)phenylimino)methyl)phenyl nicotinate* (**A8**). Yield: 91.0%; mp. 129.0 °C, FTIR (ύ, cm^−1^): 2931–2849 (CH_2_ stretching), 1735 (C=O), 1607 (C=C), 1473 (C-OAsym), 1268 (C–OSym). ^1^H-NMR (400 MHz, CDCl3) δ (ppm) 10.05 (s, 1H, CH=N), 9.59 (s, 1H, pyr), 9.08 (m, 2H, pyr), 8.11–8.05 (m, 3H, pyr, ArH), 7.41 (d, *J* = 8.5 Hz, 2H, ArH), 7.01 (d, *J* = 8.1 Hz, 2H, ArH), 6.80 (d, *J* = 8.1 Hz, 2H, ArH), 9.93 (t, *J* = 6.2 Hz, 2H, CH_3_(CH_2_)_3_CH_2_CH_2_), 1.91–1.76 (m, 2H, CH_3_(CH_2_)_3_CH_2_CH_2_), 1.56–1.51 (m, 6H, CH_3_(CH_2_)_3_CH_2_CH_2_), 0.94 (t, *J* = 6.3 Hz, 3H, CH_3_(CH_2_)_3_CH_2_CH_2_). Elemental analyses: Found (Calc.): C, 75.30 (75.32); H, 7.00 (7.02); N, 6.51 (6.51).

*(Hexadecyloxy)phenylimino)methyl)phenyl nicotinate* (**A16**). Yield: 92.7%; mp. 122.0 °C, FTIR (ύ, cm^−1^): 2921–2850 (CH_2_ stretching), 1727 (C=O), 1599 (C=C), 1473 (C-OAsym), 1268 (C–OSym). ^1^H-NMR (400 MHz, CDCl3) δ (ppm) 10.05 (s, 1H, CH=N), 9.57 (s, 1H, pyr), 9.11–9.06 (m, 2H, pyr), 8.08 (m, 3H, pyr, ArH), 7.43 (d, *J* = 8.5 Hz, 2H, ArH), 7.02 (d, *J* = 8.1 Hz, 2H, ArH), 6.80 (d, *J* = 8.1 Hz, 2H, ArH), 9.93 (t, *J* = 6.2 Hz, 2H, CH_3_(CH_2_)_3_CH_2_CH_2_), 1.94–1.76 (m, 2H,CH_3_(CH_2_)_3_CH_2_CH_2_), 1.5–1.50 (m, 6H, CH_3_(CH_2_)_3_CH_2_CH_2_), 0.93 (t, *J* = 6.3 Hz, 3H, CH_3_(CH_2_)_3_CH_2_CH_2_). Elemental analyses: Found (Calc.): C, 77.44 (77.45); H, 8.51 (8.54); N, 5.14 (5.16).

Nearly identical FT-IR spectra were observed for all investigated **An** series (Figures S3–S5, Supplementary Materials) that may be attributed to the length of the terminal alkoxy chain and does not significantly affect the location of the main characteristic FT-IR absorption bands of the investigated compounds. The effect on the C=O moiety with the slight change in the polarity character of the terminal chains was in the range of 8.0 cm^−1^ (1727 cm^−1^ for **A16**, 1729 cm^−1^ for **A6** and 1729 cm^−1^ for **A8**), while that for the C=N was 16 cm^−1^ (1591–1607 cm^−1^). It can be concluded that the little change of the polarity of the terminal alkoxy chains has a small effect on the polarization of the ester C=O and –CH=N– groups.

## 4. Conclusions

Series based on three-ring azomethine liquid crystal derivatives, 4-(4-(alkoxy)phenylimino)methyl)phenyl nicotinate (**An**), were synthesized and evaluated by experimental and computational approaches. Molecular structures were elucidated via elemental analyses, FT-IR and NMR spectroscopy. Mesomorphic characterization of its behavior was conducted by DSC, POM. Theoretical simulation of geometrical structure investigations was carried out by the DFT calculation method.

The study revealed that:

1. Independent on the terminal alkoxy chain length, all synthesized groups (**An**) are monomorphic exhibiting enantiotropic wide nematic thermal stability.
2. The structural parameters, dipole moment and the polarizability of the present compounds are highly affected by the length of the attached terminal flexible chain as well as the N atom in the heterocyclic terminal ring.
3. Binary phase diagrams constructed between different homologs showed to possess low melting temperature with wide N mesophase at the eutectic composition.
4. The different extent of the physical and structural parameters are sharing together to impact the N temperature range and the thermal stability of present compounds in their pure and mixed states.

## Figures and Tables

**Table 1:** Phase transition temperatures (**T** °C), enthalpy of transition, ΔH, kJ/mol, normalized entropy, **ΔS**, and mesophase temperature range, **ΔT**, for groups **An**.

Comp.	T_Cr-N_	ΔH_Cr-N_	T_N-I_	ΔH_N-I_	ΔS_N-I_	ΔT
**A6**	130.4	37.59	184.5	1.94	0.51	54.10
**A8**	128.6	43.40	174.2	1.80	0.48	45.60
**A16**	122.2	55.25	146.6	1.66	0.48	24.40

Cr-N denotes transition from solid to the nematic mesophase. N-I denotes transition from nematic to the isotropic mesophase.

**Table 2:** ZPE, thermal energy, enthalpy, Gibb’s free energy and entropy of the series **An** at room temperature calculated at B3LYP/6-311g(d,p) level.

Comp.	ZPE, kcal/mol	Thermal Energy, kcal/mol	Enthalpy, kcal/mol	Gibb’s Free Energy, kcal/mol	Entropy, cal/molK
**A6**	288.066	305.566	306.158	247.348	197.254
**A8**	323.766	342.923	343.515	280.483	211.414
**A16**	466.039	492.042	492.635	412.108	270.094

**Table 3:** HOMO energy, LUMO energy, orbital energy gap, ionization energy, electron affinity, dipole moment and polarizability calculated at B3LYP/6-311g(d,p) level.

Comp.	E_HOMO_, eV	E_LUMO_, eV	∆E, eV	I.E, eV	E.A, eV	Dipole Moment, Debye	Polarizability, Bohr
**A6**	−5.669	−2.097	3.572	5.669	2.097	0.896	354.830
**A8**	−5.667	−2.096	3.571	5.667	2.096	0.911	379.780
**A16**	−5.664	−2.079	3.586	5.664	2.079	0.800	477.790

## Supplementary Materials

The following are available online, Figure S1, DSC thermograms of **A6** derivative upon second heating/cooling scan with rate 10 °C/min; Figure S2, DSC thermograms of **A16** derivative upon second heating/cooling scan with rate 10 °C/min; Figure S3, FT-IR spectrum of **A6** derivative; Figure S4, FT-IR spectrum of **A8** de-rivative; Figure S5, FT-IR spectrum of **A16** derivative.
