# Supplementary material for: Synthesis, Mesomorphic and Computational Characterizations of Nematogenic Schiff Base Derivatives in Pure and Mixed State
Source: Molecules. 2021 Apr 2;26(7):2038. doi: 10.3390/molecules26072038 (PMC8038214; doi:10.3390/molecules26072038)
Supplement: Supplementary file 1 [file molecules-26-02038-s001.pdf]

## Supplementary data

# Synthesis, Mesomorphic and Computational characterizations of Nematogenic Schiff base derivatives in Pure and Mixed state

**Laila A. Al-Mutabagani[1], Latifah A. Alshabanah[1], Hoda A. Ahmed[2,3]*, Hafsa H. Alalawy[2] and Mayada H. Al alwani[3]**

[1] Chemistry Department, College of Science, Princess Nourah bint Abdulrahman University, Riyadh 11671, Saudi Arabia; (L.A.A.M) Laalmutbagani@pnu.edu.sa, (L.A.A.) Laalsabanah@pnu.eud.sa
[2] Faculty of Science, Department of Chemistry, Cairo University, Cairo 12613, Egypt
[3] Chemistry Department, College of Sciences, Yanbu, Taibah University, Yanbu 30799, Saudi Arabia
* Correspondence: Hoda A. Ahmed; ahoda@sci.cu.edu.eg

### 1. *Materials*

4-hexyloxyaniline, 4-(octyloxy)aniline, 4-(hexadecyloxy)aniline, 4-hydroxybenzaldehyde, nicotinic acid, dichloromethane and ethanol were purchased from Sigma Aldrich (Germany).

### 2. *Synthesis of 4-((4-alkoxyphenylimino))methyl)phenol (I)*

A mixture of 4-alkoxyaniline derivatives (4.1 mmol) and 4-hydroxybenzaldehyde (4.1 mmol) were dissolved in ethanol (10 ml). The solution was heated under reflux for two hours, and then allowed to cool at room temperature for complete precipitation. The obtained precipitate was filtered and washed with cold ethanol and recrystallized from hot ethanol .

### 3. *Synthesis of 4-(4-(alkoxy)phenylimino)methyl)phenyl nicotinate (An).*

A mixture of 4-dimethylaminopyridine (DMAP) (traces amount) and N, N′-dicyclohexylcarbodiimide (DCC, 0.02 mole) was added to a solution of 0.01 mole 4-((4-alkoxyphenylimino))methyl)phenol (**I**) and nicotinic acid (1.23 g, 0.01 mole) in 25 ml dry methylene chloride. The reaction mixture was kept under stirring at room temperature for 72 hours. Separated byproduct,

N,N-dicyclohexylurea, was filtered off. The filtrate was then evaporated till dryness. The obtained solid product was purified by recrystallization for twice from ethanol (**Scheme 1**).

### 4. *Characterization*

Melting points were determined by MEL-TEMP II melting point apparatus in open glass capillaries and were uncorrected. The IR spectra were recorded as potassium bromide (KBr) discs on a Perkin-Elemer FT-IR (Fourier-Transform Infrared Spectroscopy), college of Science, Taibah University. The NMR spectra were carried out at ambient temperature (~25 °C) on a (JEOL) 500 MHz spectrophotometer using tetra methyl silane (TMS) as an internal standard, NMR Unit, Faculty of Science, Mansoura University. Chemical shift was recorded as δ values in parts per million (ppm), and the signals were reported as s (singlet), d (doublet), t (triplet) and m (multiplet). Elemental analyses were analyzed at the Micro analytical Unit, Faculty of Science, Cairo University.

TA Instruments Co. (Q20 Differential Scanning Calorimeter, DSC; USA) was used for recording phase transitions. DSC calibration was carried using lead and indium melting temperature and enthalpy. Samples of 2–3 mg were used in aluminum pans for DSC investigation. The heating rate was 10°C/min in nitrogen gas as an inert atmosphere (30 ml/min). All transitions measured were for the second heating scan.

Transition temperatures for the prepared compounds were checked and phases identified by Polarized optical microscope (POM, Wild, Germany) attached with Mettler FP82HT hot stage.

### 5. *Computational characterizations*

Optimized geometries of **An** derivatives were calculated using the Gaussian 09 package [1]. Calculations were carried out using DFT approach at B3LYP/6-311G(d,p) level of theory. Frequency calculations showed that all structures were stationary points in the geometry optimization with no imaginary frequency. Ionization energy (**I.E**) is calculated as the negative values of the energy of the HOMO. **ΔE**, the energy gap, is calculated as the difference between $E_{LUMO}$ and $E_{HOMO}$. Both the Frontier molecular orbitals and the molecular electrostatic potential surfaces were generated from the formatted check (.Fchk) file of the optimized structures.

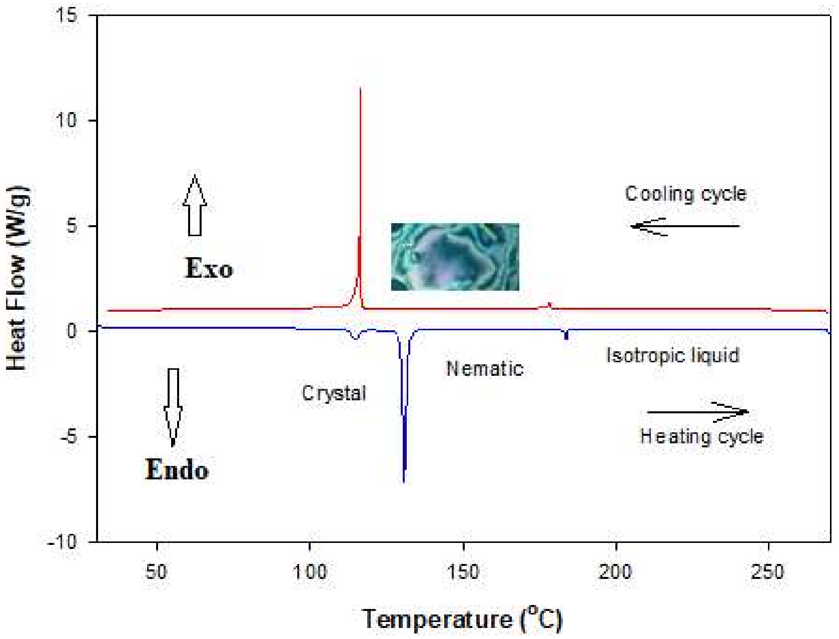


**Figure S1:** DSC thermograms of **A6** derivative upon second heating/cooling scan with rate 10 °C /min.

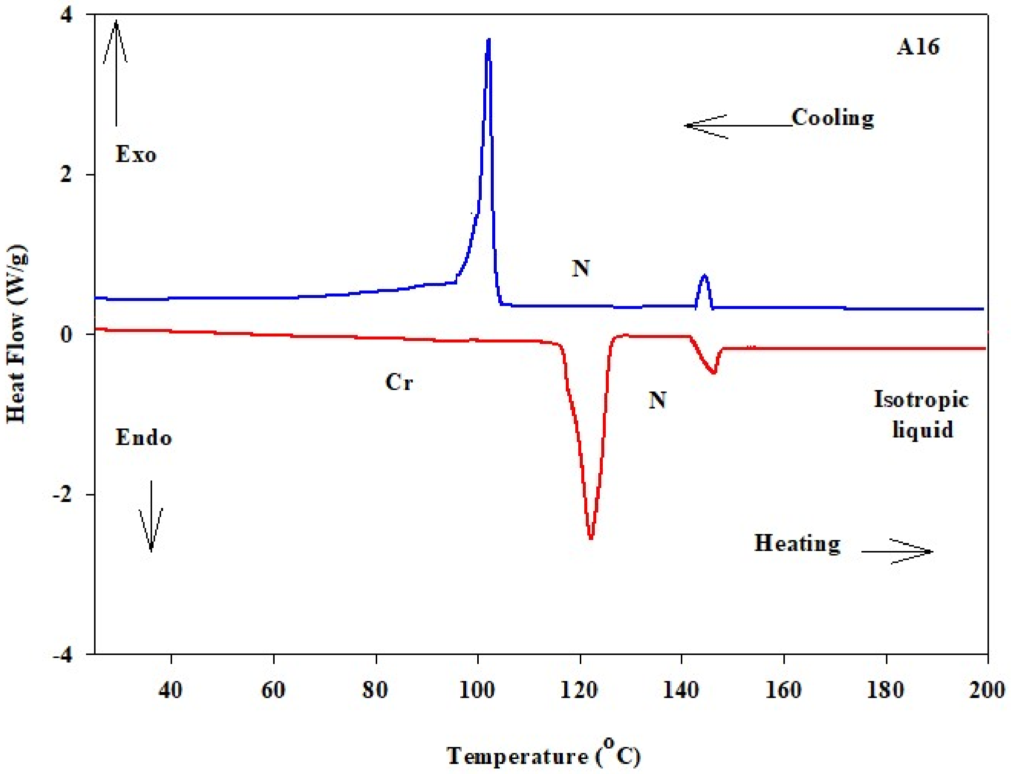


**Figure S2:** DSC thermograms of **A16** derivative upon second heating/cooling scan with rate 10 °C /min.

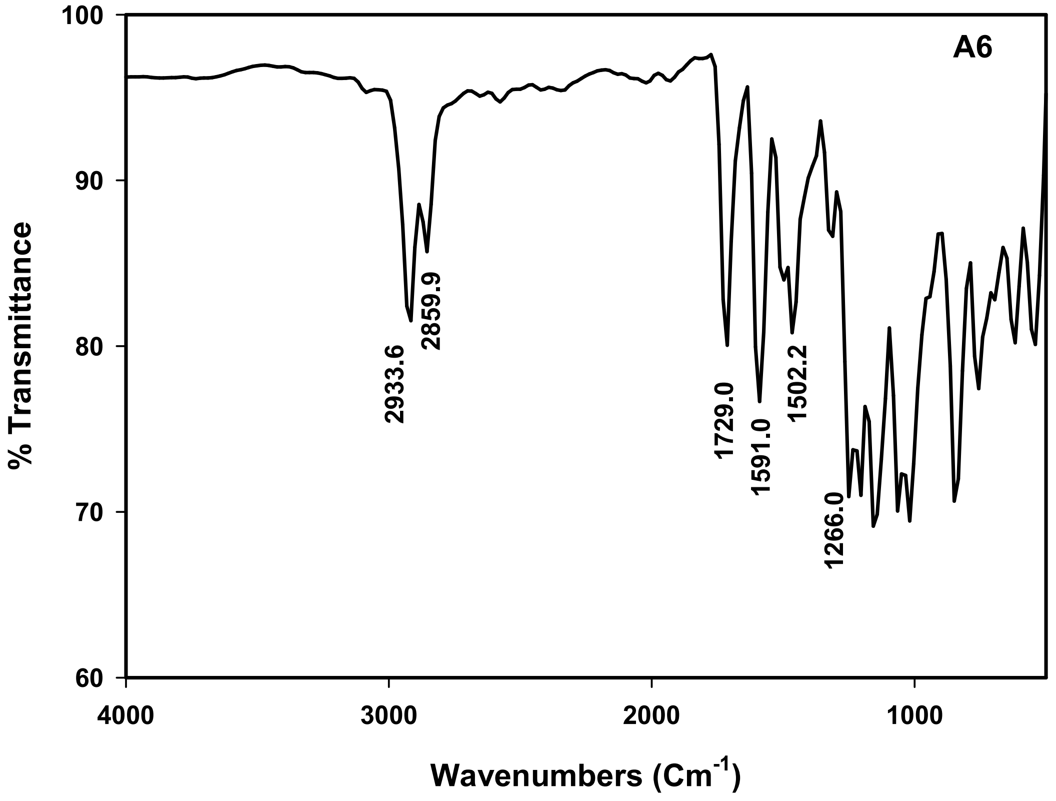


**Figure S3:** FT-IR spectrum of **A6** derivative.

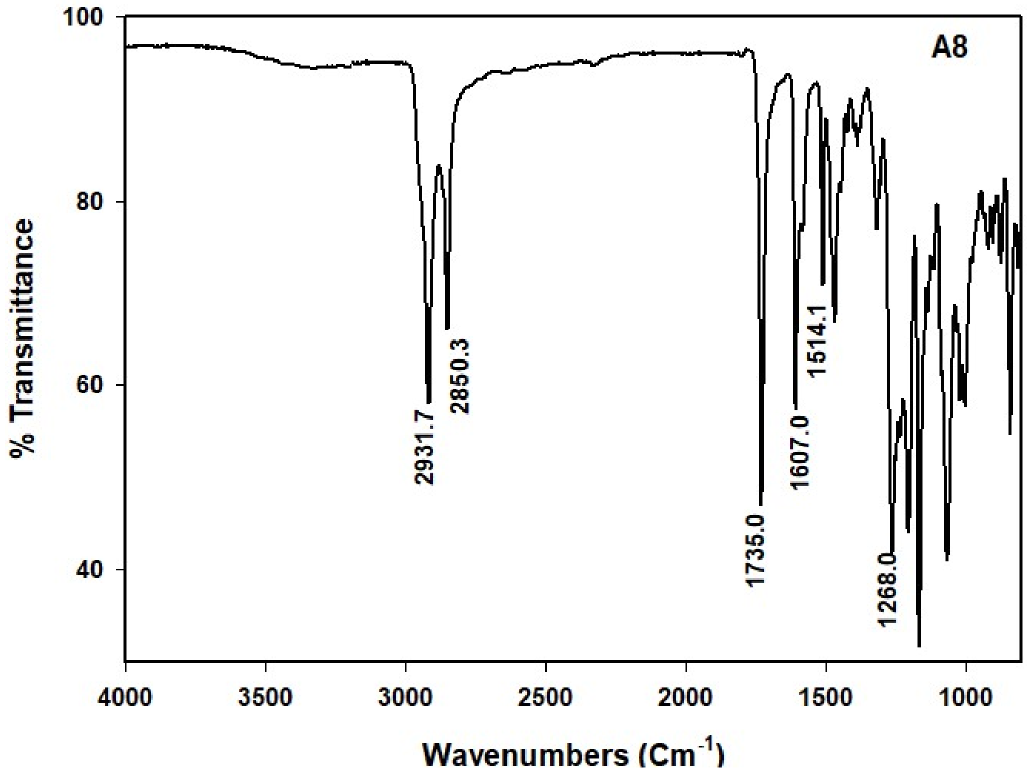


**Figure S4:** FT-IR spectrum of **A8** derivative.

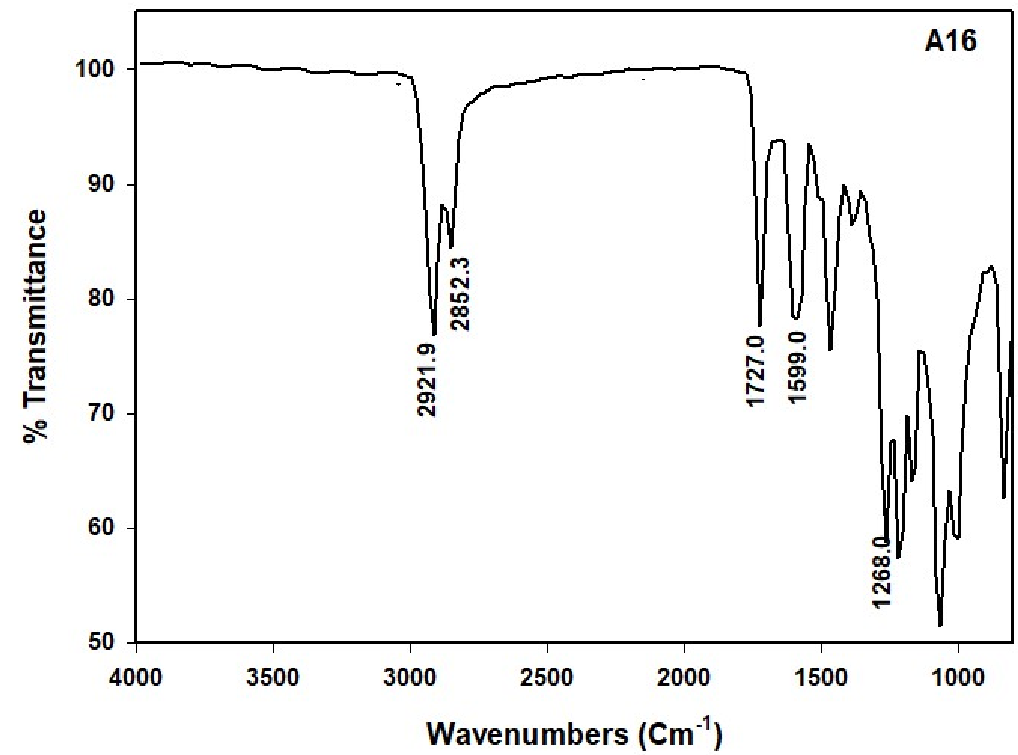


**Figure S5:** FT-IR spectrum of **A16** derivative.

**References:**

1. M. J. Frisch, G. W. Trucks, H. B. Schlegel, G. E. Scuseria, M. A. Robb, J. R. Cheeseman, G. Scalmani, V. Barone, B. Mennucci, G. A. Petersson, H. Nakatsuji, M. Caricato, X. Li, H. P. Hratchian, A. F. Izmaylov, J. Bloino, G. Zheng, J. L. Sonnenberg, M. Hada, M. Ehara, K. Toyota, R. Fukuda, J. Hasegawa, M. Ishida, T. Nakajima, Y. Honda, O. Kitao, H. Nakai, T. Vreven, J. A. Montgomery Jr., J. E. Peralta, F. Ogliaro, M. Bearpark, J. J. Heyd, E. Brothers, K. N. Kudin, V. N. Staroverov, R. Kobayashi, J. Normand, K. Raghavachari, A. Rendell, J. C. Burant, S. S. Iyengar, J. Tomasi, M. Cossi, N. Rega, J. M. Millam, M. Klene, J. E. Knox, J. B. Cross, V. Bakken, C. Adamo, J. Jaramillo, R. Gomperts, R. E. Stratmann, O. Yazyev, A. J. Austin, R. Cammi, C. Pomelli, J. W.

Ochterski, R. L. Martin, K. Morokuma, V. G. Zakrzewski, G. A. Voth, P. Salvador, J. J. Dannenberg, S. Dapprich, A. D. Daniels, Ö. Farkas, J. B. Foresman, J. V Ortiz, J. Cioslowski, and D. J. Fox, Gaussian 09, Revision A.02, Gaussian Inc Wallingford CT, Gaussian, Inc., Wallingford CT, (2009).
